# Comparative proteomic analysis of the telogen-to-anagen transition in cashmere goat secondary hair follicles

**DOI:** 10.3389/fvets.2025.1542682

**Published:** 2025-02-25

**Authors:** Xiaoyu Han, Guizhen Gao, Nanxiang Sun, Bai Dai, Liqing Ren, Haobing Bai, Jianing Liu, Jie Liu, Hongyan Zhao, Dongjun Liu

**Affiliations:** ^1^Key Laboratory of Reproductive Regulation and Breeding of Grassland Livestock, School of Life Sciences, Inner Mongolia University, Hohhot, China; ^2^Peking University Cancer Hospital, Affiliated Cancer Hospital of Inner Mongolia Medical University, Hohhot, China; ^3^Reproductive Medicine Center, the Affiliated Hospital of Inner Mongolia Medical University, Hohhot, China; ^4^Inner Mongolia People’s Hospital NHC Key Laboratory of Diagnosis and Treatment of COPD, Inner Mongolia Key Laboratory of Respiratory Diseases, Hohhot, China; ^5^Tongliao Institute of Agriculture and Animal Husbandry, Tongliao, China; ^6^Agriculture and Animal Husbandry Technology Extension Center, Etuoke Banner, China

**Keywords:** cashmere goat secondary hair follicles, telogen-to-anagen transition, proteomic analysis, ADAM17, Sfrp1, PPP1CA, secondary hair follicle cycle regulation

## Abstract

Secondary hair follicles (SHFs) in cashmere goats produce high-value cashmere fibers, which cyclic regulation is critical for optimizing cashmere yield and quality. This study explores the phenotypic changes and differential protein expression profiles involved in the telogen-to-anagen transition of SHFs. Through histological observations, proteomic analyses, and immunohistochemical validation, we identified key molecular features and regulatory pathways underlying SHF cyclic renewal. Histological analysis showed that telogen-phase SHFs exhibit a reduced volume, decreased dermal papilla cell (DPC) and hair matrix cell (HMC) activity, compact structure, and superficial localization in the dermis. Anagen-phase SHFs exhibit significantly increased volume, deeper dermal penetration, and active cell proliferation. Proteomic analysis identified 3,654 proteins in skin samples, with 458 differentially expressed proteins (DEPs) significantly associated with biological processes such as cell adhesion, signal transduction, protein synthesis, and metabolism. These DEPs were enriched in key regulatory pathways, including Notch, Wnt, Jak–STAT, PI3K-Akt, and ECM-receptor interaction. Protein–protein interaction analysis identified A Disintegrin and Metalloproteinase Domain 17 (ADAM17), Secreted Frizzled-Related Protein 1 (SFRP1), and Protein Phosphatase 1 Catalytic Subunit Alpha (PPP1CA) as core regulators of SHF cyclic transitions. Validation by RT-qPCR, Western blot, and immunohistochemical analyses confirmed that ADAM17, SFRP1, and PPP1CA were predominantly localized in functional regions, including the outer root sheath (ORS), dermal papilla (DP), and hair matrix (HM). Their expression levels were significantly enhanced during anagen. ADAM17 is suggested to promote the growth of SHFs by regulating ORS cells proliferation and mediating signal transduction in DPCs, while SFRP1, as a modulator of the Wnt signaling pathway, likely supports SHFs growth and regeneration by modulating the activity of Secondary hair follicle stem cells (SHFSCs) and promoting the differentiation of HMCs. PPP1CA may enhance cell proliferation and metabolic activity by modulating phosphorylation states. In conclusion, this study identifies key molecular factors and pathways driving the telogen-to-anagen transition in cashmere goat SHFs. It emphasizes the roles of ADAM17, SFRP1, and PPP1CA in SHF renewal and offers insights into SHF development mechanisms and cashmere fiber improvement.

## 1 Introduction

SHFs in cashmere goats produce cashmere, a highly valuable natural fiber renowned for its exceptional softness, warmth, and lightness. The growth and shedding of these fibers are tightly regulated by the cyclical activity of SHFs, alternating between telogen and anagen phases (1). Understanding the molecular mechanisms that govern the transition of the SHF cycle is essential for optimizing both cashmere yield and quality.

The hair follicle cycle comprises three distinct phases: anagen, catagen, and telogen. This finely tuned process is orchestrated by the complex interplay of multiple signaling pathways, which regulate the activation of hair follicle stem cells (HFSCs), the function of DPCs, and the dynamic remodeling of the extracellular matrix (ECM). These mechanisms collectively control the transitions between different phases of the follicular cycle (2–4). Among these pathways, the Wnt signaling pathway plays a central role by activating HFSCs to drive the transition from telogen to anagen, while its dysregulation can impair follicular regeneration (5–8). The Notch signaling pathway maintains the balance between HFSC self-renewal and differentiation (9), working synergistically with Wnt signaling to ensure proper follicular regeneration (10). Dysregulated Notch signaling can delay follicular cycle progression (11). The Ras-MAPK signaling pathway, particularly through its ERK and p38 branches, regulates the proliferation and differentiation of DPCs, making it critical for the anagen phase (12–14). Meanwhile, the ECM-receptor interaction pathway sustains follicular microenvironment homeostasis by providing structural support and transmitting biochemical signals, playing a vital role in cycle transitions (15). The Jak–STAT signaling pathway mediates cytokine-driven signal transduction to maintain the quiescent state of hair follicles, and its inhibition facilitates the induction of hair growth from dormant follicles (16, 17). Additionally, the Hippo pathway regulates the proliferation and differentiation of HFSCs through its downstream effectors YAP/TAZ (18). The PI3K-Akt pathway supports anagen by regulating cell survival, proliferation, and metabolism (19), while the TNF signaling pathway promotes the transition from telogen to anagen by activating HFSCs through the AKT/*β*-catenin pathway (20). Despite advancements in understanding the roles of these signaling pathways in hair follicle cycling, the specific molecular regulators and their dynamic expression patterns during the telogen-to-anagen transition in the SHFs of cashmere goats remain poorly understood.

This study investigates the phenotypic and molecular changes underlying the telogen-to-anagen transition in cashmere goat SHFs. By integrating histological observations, proteomic analyses, and the validation and localization of key proteins, we comprehensively uncover the dynamic regulatory mechanisms governing the cycling of SHFs. Specifically, this study highlights the critical roles of ADAM17, SFRP1, and PPP1CA in regulating the telogen-to-anagen transition and promoting the growth of cashmere fibers. These findings enhance our understanding of hair follicle biology and provide a theoretical foundation for improving cashmere production through molecular breeding or biotechnological approaches.

## 2 Materials and methods

### 2.1 Experimental animals and sample collection

Six healthy adult cashmere goats (*n* = 6, aged 1 year; 3 females and 3 males) were selected from the Yiwei White Cashmere Goat Farm in Ordos, Inner Mongolia, China. All animals were maintained under standard feeding conditions with unrestricted access to food and water. Skin tissue samples were collected from the dorsal, flank, and abdominal regions during the telogen phase (April) and anagen phase (September) of the SHF cycle. These regions were chosen because they represent the primary distribution areas of SHFs and exhibit significant differences in density, developmental state, and cycle activity. The dorsal region has the highest SHF density and the most active anagen-phase follicles, making it the primary source of high-quality cashmere fibers. The flank region shows intermediate follicle density and activity, with structural characteristics between those of the dorsal and abdominal regions. The abdominal region has the lowest follicle density, and some SHFs may be in a degenerated state. Sampling these regions provides a comprehensive representation of the periodic changes in SHFs across the goat’s body and offers a basis for exploring region-specific regulatory mechanisms. The phases of the SHF cycle were determined through histological examination and follicular morphology assessment. The collected skin samples (~1 cm^2^) were immediately divided into two parts: one part was fixed in 4% paraformaldehyde for histological and immunohistochemical analyses, while the other part was rapidly frozen in liquid nitrogen for RNA and protein extraction, proteomic sequencing, and subsequent analyses.

### 2.2 Experimental design

**Figure 1:**
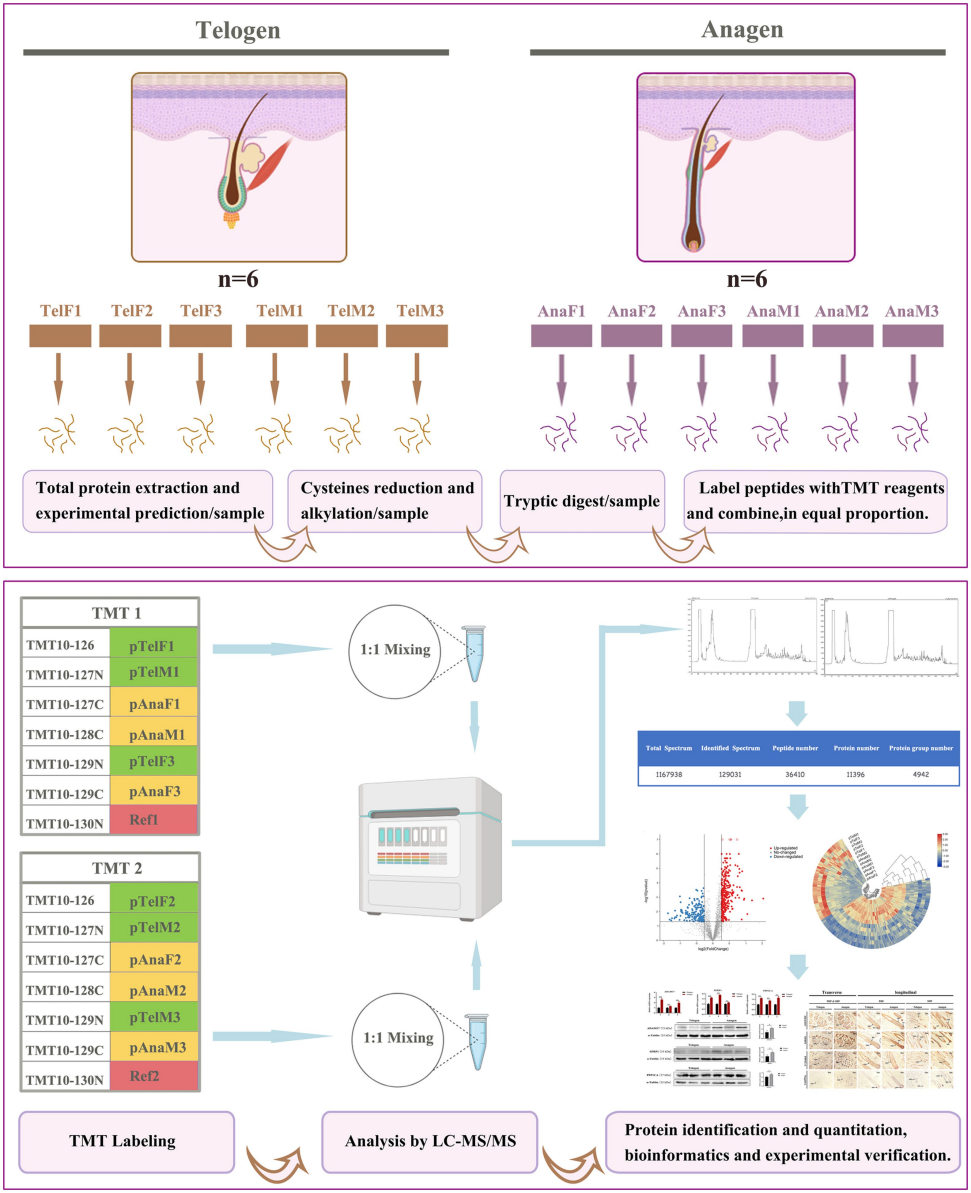
Workflow of TMT-labeled proteomics analysis of SHFs during the telogen- and anagen-phase transitions. This study utilized TMT labeling combined with LC–MS/MS to identify and quantify the proteome of cashmere goat SHFs during the telogen and anagen phases. Skin tissue samples included six telogen samples (three females and three males) and six anagen samples (the same three females and three males). After protein extraction, cysteine reduction, and alkylation treatment, the proteins were digested into peptides and labeled with TMT reagents. The labeled samples were mixed in a 1:1 ratio and analyzed via LC–MS/MS for protein identification and quantification. DEPs were further analyzed using bioinformatics to elucidate their functions, while the expression patterns and localization of key proteins were experimentally validated.

As shown in Figure 1, this study employed tandem mass tag (TMT)-based labeling technology combined with LC–MS/MS to identify and quantify the proteome of cashmere goat SHFs during the telogen and anagen phases. Skin tissue samples from six goats were used: three females (TelF1, TelF2, TelF3) and three males (TelM1, TelM2, TelM3) during telogen, and the same three females (AnaF1, AnaF2, AnaF3) and three males (AnaM1, AnaM2, AnaM3) during anagen. Samples were divided into two independent experimental groups for analysis. Proteins were first extracted from skin at both telogen and anagen phases. Samples underwent reduction and alkylation of cysteine residues to minimize disulfide bond interference. Proteins were then digested into peptides using trypsin. The peptide samples were labeled with TMT reagents (TMT10plex), with TMT tags assigned to telogen and anagen samples in each experiment, alongside a reference sample (Ref) composed of an equal mix of all experimental samples. The labeled samples were mixed at a 1:1 ratio for subsequent analysis. The mixed samples were analyzed using liquid chromatography–tandem mass spectrometry (LC–MS/MS) for protein identification and quantification. Mass spectrometry (MS) data were used to generate total spectra, identified spectra, peptide counts, and the protein dataset. Proteomic data were subjected to statistical analysis to identify DEPs, which were then functionally annotated and enriched. Correlation analyses were conducted to further explore biological differences between telogen and anagen. Additionally, key DEPs were validated using real-time quantitative PCR (RT-qPCR) and Western blotting, and their expression patterns and localization were evaluated via immunohistochemistry.

### 2.3 Histological and immunohistochemical analysis

Skin samples were fixed in 4% paraformaldehyde, dehydrated, embedded in paraffin, and sectioned at a thickness of 7 μm. The sections were stained with hematoxylin and eosin (H&E) to observe SHF structure and confirm the telogen or anagen phase. For immunohistochemical analysis, paraffin sections were deparaffinized, rehydrated, and subjected to antigen retrieval in boiling sodium citrate buffer. Endogenous peroxidase activity was blocked with 3% hydrogen peroxide. Sections were incubated overnight at 4°C with primary antibodies. The following day, the sections were incubated with HRP-conjugated secondary antibodies for 1 h, followed by DAB staining. Images were captured using an optical microscope (Nikon, Tokyo, Japan). Detailed antibody information is provided in Supplementary Table S1.

### 2.4 RNA extraction and RT-qPCR

Total RNA was extracted from skin samples using RNAiso reagent (Takara Bio Inc., Shiga, Japan) following the manufacturer’s protocol. RNA purity and concentration were measured using a NanoDrop Spectrophotometer (Thermo Fisher Scientific, Waltham, MA, United States). Complementary DNA (cDNA) was synthesized using the PrimeScript FAST reverse transcription (RT) reagent kit with genomic DNA (gDNA) eraser (Takara Bio Inc., Shiga, Japan). Reverse transcription quantitative real-time polymerase chain reaction (RT-qPCR) was performed on a CFX96 Real-Time PCR System (Bio-Rad Laboratories, Hercules, CA, United States) with TB Green® Premix Ex Taq™ II (Takara Bio Inc., Shiga, Japan). Specific primers were designed for ADAM17, SFRP1, and PPP1CA, with GAPDH as the reference gene for relative expression calculation using the 2^-ΔΔCt method. Primer sequences are listed in Supplementary Table S2.

### 2.5 Protein extraction and western blot

Total protein was extracted from skin samples using a Mammalian Protein Extraction Kit (CWBIO, Beijing, China) according to the manufacturer’s instructions. Protein concentration was determined using a BCA Protein Assay Kit (Thermo Fisher Scientific, Waltham, WA, United States). Equal amounts of protein (20 μg per lane) were separated by SDS-PAGE and transferred to PVDF membranes. Membranes were blocked with 5% non-fat milk at room temperature for 1 h and incubated overnight at 4°C with primary antibodies. After incubation with HRP-conjugated secondary antibodies for 1 h, protein bands were visualized using the Tanon 5,200 Imaging System (Tanon, Shanghai, China). Band intensity was quantified using ImageJ software, and statistical analysis was performed to compare protein expression levels across groups. Detailed antibody information is provided in Supplementary Table S3.

### 2.6 Proteomic sequencing and bioinformatic analysis

Following protein extraction, protein solutions were reduced with 10 mmol/L TCEP at 37°C for 1 h and alkylated with 40 mmol/L iodoacetamide at room temperature in the dark for 30 min. Trypsin (Promega, Madison, WI, United States) was added at an enzyme-to-protein ratio of 1:50 (w/w) for overnight digestion at 37°C. The resulting peptides were labeled with TMT reagents (Thermo Fisher Scientific, Waltham, MA, United States) and incubated at room temperature for 2 h, followed by a 15-min reaction with hydroxylamine. Equal amounts of labeled peptides were mixed, vacuum-dried, re-dissolved in 0.1% formic acid, and analyzed using a Thermo Scientific Q Exactive mass spectrometer coupled with an EASY-nLC 1,200 nano-liquid chromatography system.

Peptide samples were loaded onto a C18 column (75 μm × 25 cm, Thermo Fisher Scientific, Waltham, MA, United States) and separated using gradient elution with acetonitrile (ACN) containing 0.1% formic acid at a flow rate of 300 μL/min. The mass spectrometer was operated in data-dependent acquisition (DDA) mode, with a full scan range of 350–1,300 m/z at resolutions of 70,000 (full scan) and 35,000 (MS/MS scan), followed by MS/MS analysis of the top 20 most abundant precursor ions. Raw mass spectrometry data were analyzed using Proteome Discoverer™ Software 2.2 against the UniProt goat protein database. The false discovery rate (FDR) for peptide and protein identification was set to ≤1%, with proteins identified by at least one unique peptide.

TMT labeling technology, with the quantification based on the intensity of the reporter ions. To correct for potential technical variances, normalization of the protein expression levels was conducted across samples using total ion intensity normalization. Additionally, inter-TMT batch normalization was performed using an internal reference sample, ensuring consistent protein quantification across independent TMT experiments. This step minimized batch effects and allowed for accurate comparison of protein expression levels across all samples.

Differential protein expression was analyzed using the *R* statistical package with the *t*-test function. DEPs were defined as upregulated [*p* < 0.05 and fold change (FC) > 1.2] or downregulated (*p* < 0.05 and FC < 0.83). Heatmaps of DEPs were generated using the R package pheatmap. Functional annotation of DEPs was performed using Gene Ontology (GO), while Kyoto Encyclopedia of Genes and Genomes (KEGG) enrichment analysis was conducted using the clusterProfiler R package. Protein–protein interaction (PPI) networks were constructed using the STRING database and visualized with Cytoscape software.

### 2.7 Statistical analysis

All data are presented as means ± standard deviation (SD). Student’s t-test was used for significance testing between two groups, while one-way analysis of variance (ANOVA) was applied for comparisons among three or more groups. A significance threshold of *p* < 0.05 was used.

## 3 Results

### 3.1 Significant phenotypic changes observed during the telogen-to-anagen transition of SHFs

Skin samples from the dorsal, flank, and abdominal regions of cashmere goats were examined using hematoxylin and eosin (H&E) staining, along with transverse and longitudinal observations. These analyses revealed significant phenotypic changes in SHFs during the telogen-to-anagen transition, as well as notable structural differences among the regions.

**Figure 2:**
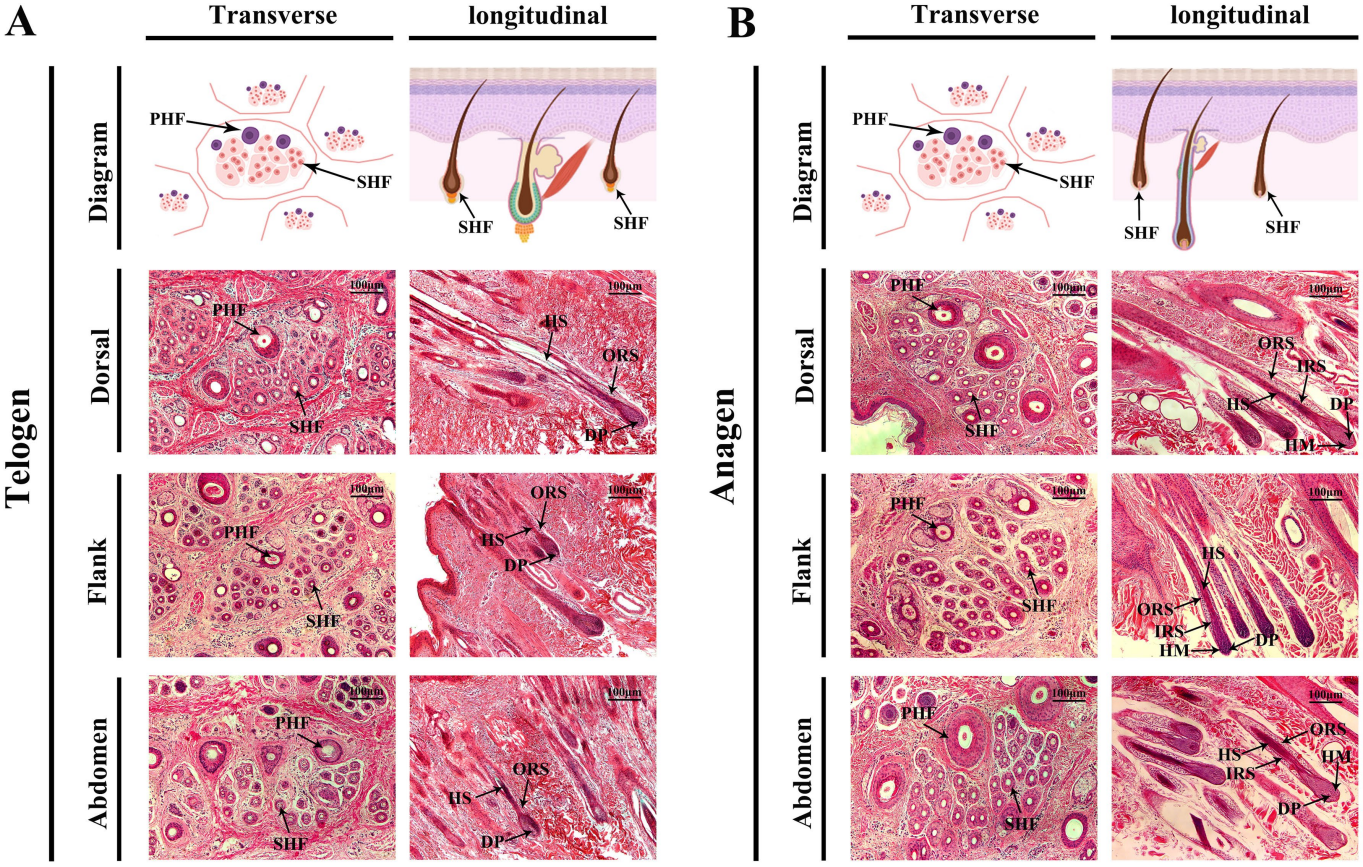
Morphological changes in cashmere goat SHFs during the telogen and anagen phases. **(A)** Representative cross-sections and longitudinal sections of SHFs in the telogen phase. Transverse sections show primary and secondary hair follicles in the dorsal, flank, and abdominal regions. Telogen-phase SHFs appear significantly shrunken, with compact, round, or oval structures. Longitudinal sections display shorter telogen-phase SHFs with their bases located close to the epidermis. The DP and HM are underdeveloped, and the overall follicular structure is degenerated. **(B)** Representative cross-sections and longitudinal sections of SHFs in the anagen phase. Transverse sections reveal enlarged SHFs in the dorsal, flank, and abdominal regions, with increased diameter and well-defined structures. Longitudinal sections demonstrate the deep extension of SHFs into the dermis, with well-developed DP, HM, IRS, and ORS. Scale bar: 100 μm.

During the telogen phase (Figure 2A), SHFs exhibited significant phenotypic changes, including a substantial reduction in follicle volume, a decrease in number, more superficial positioning within the skin, and a more compact structure. Moreover, the activity of DPCs and HMCs was markedly reduced. Transverse sections revealed that dorsal SHFs had the highest density, compact structures, and relatively well-preserved morphology; flank SHFs showed moderate density and regular morphology; while abdominal SHFs had the lowest density, larger cross-sectional areas, and more pronounced degeneration of secondary structures. Longitudinal sections further demonstrated that dorsal SHFs, despite being positioned closer to the epidermis, maintained greater depth and structural integrity. In contrast, abdominal SHFs were more superficial, with severe degeneration of secondary structures and nearly complete cessation of hair growth activity.

During the anagen phase (Figure 2B), SHFs exhibited significant phenotypic changes. The follicle volume increased substantially, structures became more distinct, and follicles extended deeper into the skin. Dorsal SHFs showed the most pronounced growth, with follicle bases reaching the deep dermis, the highest proliferative activity in HMCs, and well-developed SHFs contributing to robust hair growth. Flank SHFs displayed intermediate growth, with follicle bases extending to the mid-dermis and moderately developed structures. Abdominal SHFs exhibited the smallest increase in volume and depth, extending only to the superficial dermis. Poorly developed SHFs in this region resulted in reduced hair production. Transverse sections revealed a significant increase in the number and diameter of SHFs in the dorsal region, followed by the flank, while abdominal SHFs showed the smallest increase. Longitudinal sections further demonstrated that dorsal hair papillae and ORS structures were the most well-developed, whereas abdominal SHFs displayed relatively simple and underdeveloped structures.

In summary, the phenotypic differences between telogen and anagen SHFs were reflected in their size, position, developmental state, and cellular activity. Furthermore, SHFs in the dorsal, flank, and abdominal regions exhibited distinct structural and developmental characteristics. These region-specific features provide an important foundation for further investigations into the molecular mechanisms underlying the telogen-to-anagen transition and the functional diversity of SHFs across body regions.

### 3.2 Proteomic analysis reveals characteristic protein expression during the telogen-to-anagen transition of SHFs

To elucidate protein expression profiles during the transition of SHFs from the telogen-to-anagen phase, high-throughput proteomic analysis was performed on skin samples from cashmere goat. Telogen-phase skin samples were used as the control group, while anagen-phase samples constituted the experimental group. Each group included six biological replicates (three male and three female goats), with all samples collected from the same six goats to ensure consistency.

Following protein extraction, enzymatic digestion, liquid chromatography-mass spectrometry (LC–MS) analysis, and quality control, a total of 3,654 proteins were identified through database matching (Supplementary Table S4). These identified proteins were associated with multiple key processes related to hair follicle growth and metabolism, providing a foundation for subsequent analysis of DEPs and their roles in regulating the cycle of SHFs.

#### 3.2.1 Data correlation analysis

To evaluate the effects of different growth stages and sexes on protein expression in SHFs, principal component analysis (PCA) was performed on proteomic data from telogen-phase females (pTelF), anagen-phase females (pAnaF), telogen-phase males (pTelM), and anagen-phase males (pAnaM).

**Figure 3:**
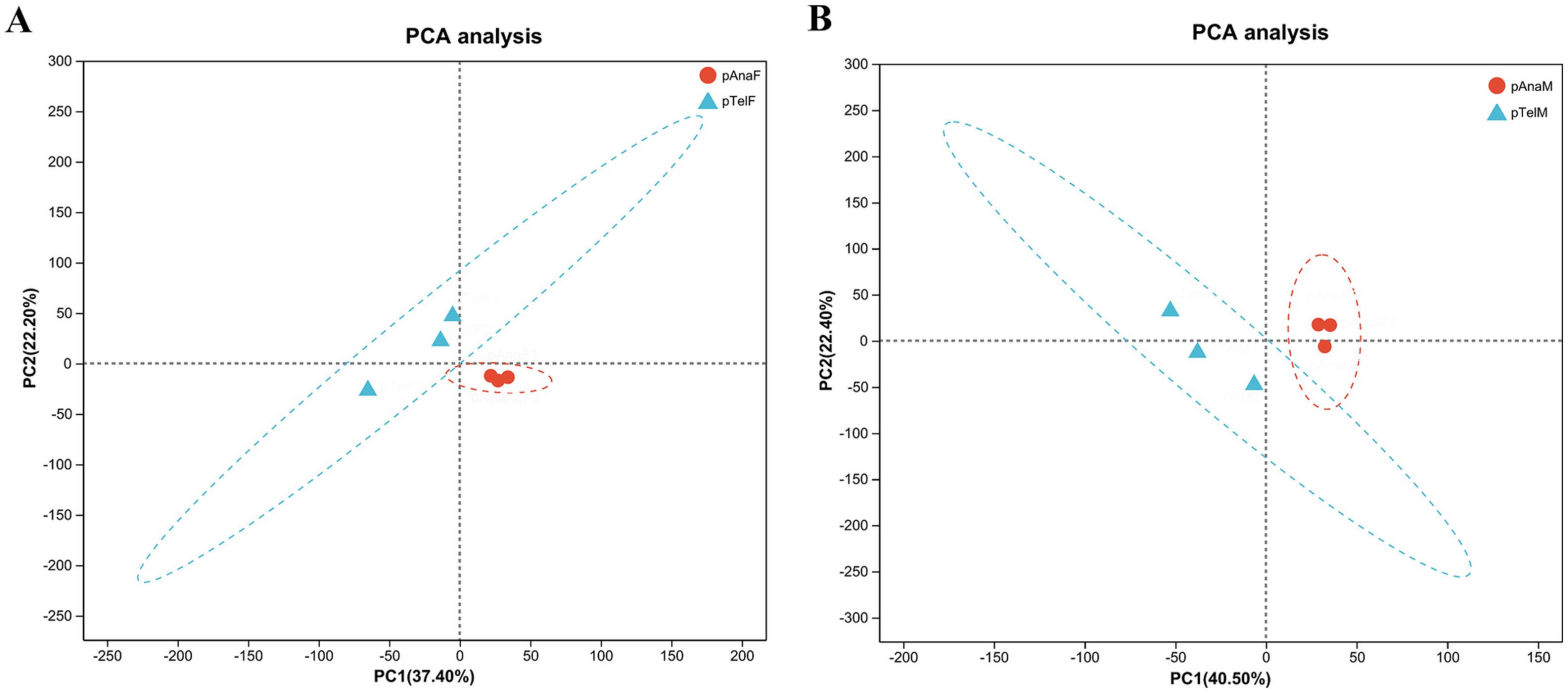
PCA of protein expression profiles in telogen- and anagen-phase SHFs of cashmere goats. **(A)** PCA of protein expression in telogen-phase (pTelF) and anagen-phase (pAnaF) female goats. PC1 (37.40%) and PC2 (22.20%) account for the majority of the variation. Telogen-phase samples (blue triangles) are distinctly separated from anagen-phase samples (red circles) along the PC1 axis. Anagen-phase samples exhibit tighter clustering, suggesting greater consistency in protein expression. **(B)** PCA of protein expression in telogen-phase (pTelM) and anagen-phase (pAnaM) male goats. PC1 (40.50%) and PC2 (22.40%) account for the majority of the variation. Telogen-phase samples (blue triangles) are clearly separated from anagen-phase samples (red circles) along the PC1 axis, with anagen-phase samples showing tighter clustering, indicating higher consistency in protein expression.

For female samples, PCA revealed that PC1 and PC2 accounted for 37.40 and 22.20% of the total variation, respectively. Telogen-phase samples (pTelF) and anagen-phase samples (pAnaF) were clearly separated along the PC1 axis, with anagen-phase samples clustering tightly. This clustering indicated high within-group consistency in protein expression during anagen. In contrast, telogen-phase samples were more dispersed, reflecting greater heterogeneity in protein expression during this phase (Figure 3A).

For male samples, PCA showed that PC1 and PC2 explained 40.50 and 22.40% of the total variation, respectively. Similarly, telogen-phase samples (pTelM) and anagen-phase samples (pAnaM) were distinctly separated along the PC1 axis. Anagen-phase male samples also clustered tightly, further supporting the observation of consistent protein expression during anagen, while telogen-phase samples exhibited greater dispersion (Figure 3B).

Overall, PCA results indicated significant differences in protein expression between telogen and anagen phases, regardless of sex. Protein expression in anagen-phase samples was more consistent, while telogen-phase samples displayed greater heterogeneity. These findings provide a foundation for further exploration of DEPs and their regulatory mechanisms in the SHF growth cycle.

#### 3.2.2 Functional annotation and enrichment analysis of DEPs

To identify changes in protein expression during the transition of SHFs from the telogen-to-anagen phase, a two-tailed Student’s t-test was performed to evaluate statistical significance, with a *p* < 0.05 considered significant. Proteins with a fold change (FC) > 1.2 were classified as upregulated, while those with an FC < 0.83 were classified as downregulated. A total of 458 DEPs were identified (Supplementary Table S5), including 293 upregulated and 165 downregulated proteins, indicating significant differences in protein expression between the two phases of SHFs.

**Figure 4:**
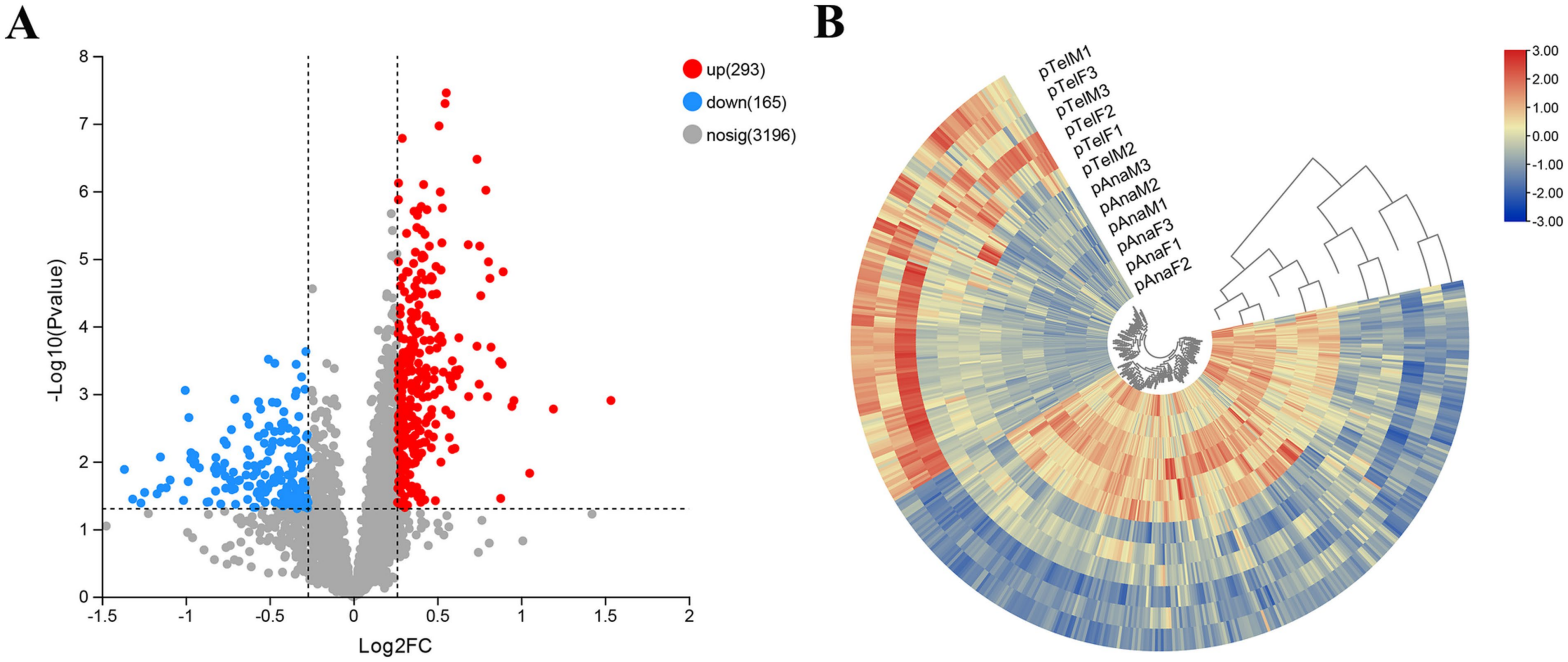
Identification and clustering of DEPs in telogen- and anagen-phase SHFs of cashmere goats. **(A)** Volcano plot illustrating the distribution of DEPs. The X-axis represents the log2(fold change) of protein expression, while the Y-axis represents the -log10(*p*-value). Proteins significantly upregulated (log2FC > 0.32, *p* < 0.05) are shown in red, whereas those significantly downregulated (log2FC < −0.26, *p* < 0.05) are shown in blue. Gray dots indicate proteins without significant expression changes. A total of 458 DEPs were identified, including 293 upregulated and 165 downregulated proteins. **(B)** Hierarchical clustering heatmap of DEPs. Samples include telogen-phase male goats (pTelM), telogen-phase female goats (pTelF), anagen-phase male goats (pAnaM), and anagen-phase female goats (pAnaF). Rows represent DEPs, while columns represent biological replicates. Red indicates upregulated proteins, blue indicates downregulated proteins, and yellow indicates proteins with no significant changes. Samples are distinctly clustered into telogen- and anagen-phase groups, highlighting differential protein expression patterns between the two phases.

A volcano plot displayed the distribution of DEPs, illustrating that numerous proteins were significantly upregulated in anagen-phase SHFs, while some were markedly downregulated (Figure 4A). A heatmap of the DEPs revealed distinct clustering patterns, indicating clear grouping trends in protein expression between the two phases of SHFs (Figure 4B).

To investigate the biological functions and signaling pathways involved in the transition from the telogen-to-anagen phase of SHFs, GO functional annotation and KEGG pathway enrichment analyses were conducted on the identified DEPs. GO analysis classified DEPs into three main categories: biological processes (BP), cellular components (CC), and molecular functions (MF).

**Figure 5:**
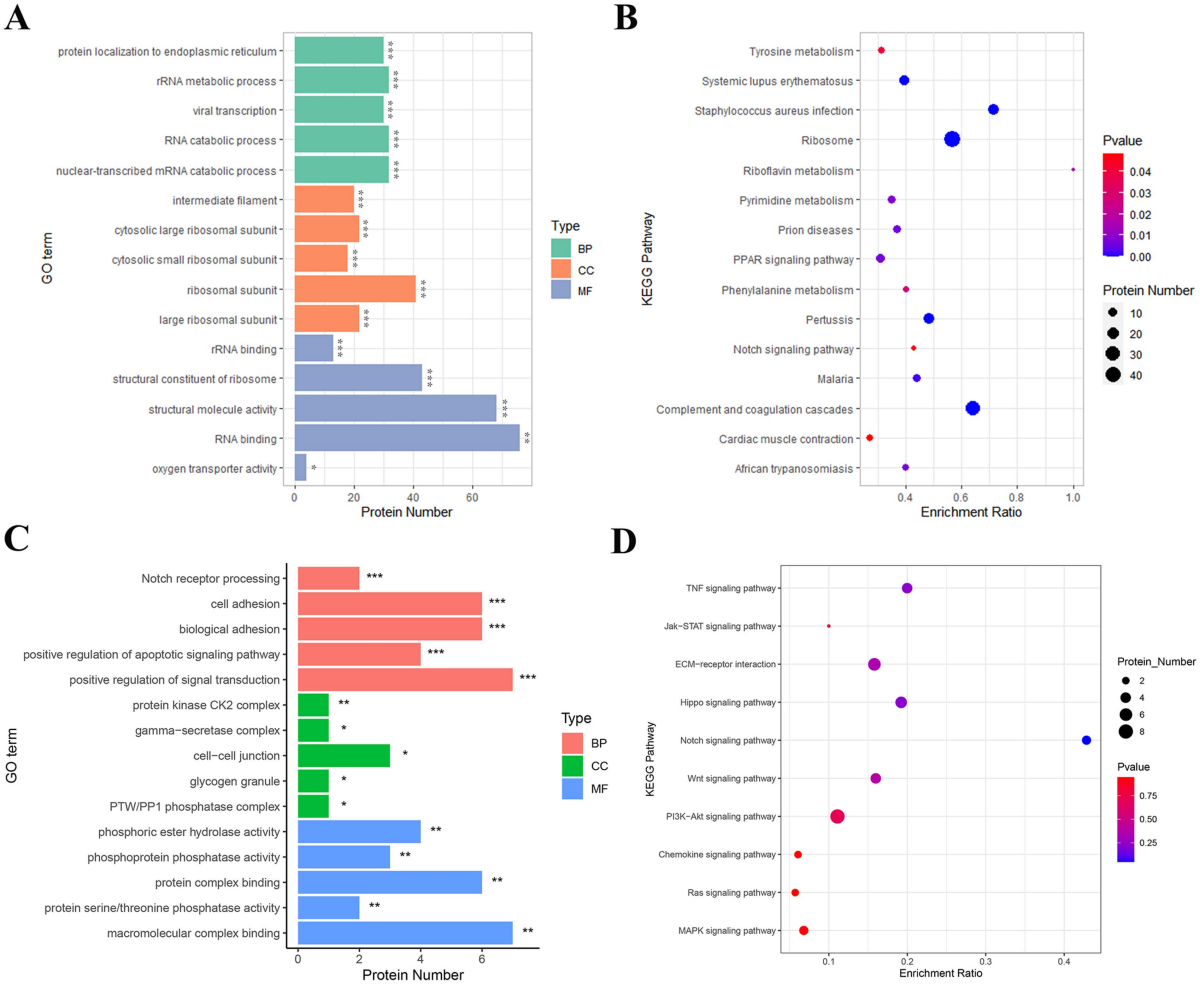
GO and KEGG enrichment analyses of key proteins involved in SHF development and regeneration. **(A)** GO enrichment analysis of the 458 DEPs identified between telogen- and anagen-phase skin samples. The most significantly enriched GO terms include RNA binding (MF), structural constituent of ribosome (MF), rRNA binding (MF), and cytosolic ribosomal subunit (CC), highlighting their roles in translation, protein synthesis, and cellular structure. **(B)** KEGG pathway analysis of the 458 DEPs. The DEPs were significantly enriched in pathways such as ribosome, pyrimidine metabolism, PPAR signaling pathway, and Notch signaling pathway, suggesting their involvement in cellular metabolic processes and hair follicle development. **(C)** GO enrichment analysis of 28 key proteins from 10 hair follicle-related pathways. These proteins were significantly enriched in terms such as cell adhesion (BP), Notch receptor processing (BP), *γ*-secretase complex (CC), and macromolecular complex binding (MF), indicating their roles in cell communication, protein complex assembly, and signal transduction during the telogen-to-anagen transition. **(D)** KEGG pathway analysis of the 28 key proteins. These proteins were significantly enriched in pathways such as Notch signaling, Wnt signaling, Hippo signaling, and ECM-receptor interaction, which are critical for hair follicle morphogenesis, regeneration, and stem cell activation.

In the BP category, DEPs were significantly enriched in processes such as rRNA metabolism, RNA catabolic processes, and protein targeting to the endoplasmic reticulum, indicating their critical roles in regulating cellular transcription and translation. In the CC category, DEPs were primarily enriched in ribosomal subunits (both large and small) and intermediate filaments, suggesting their association with cellular organelles and cytoskeletal structures essential for SHF function and morphology. In the MF category, DEPs were significantly enriched in RNA binding, structural molecule activity, and oxygen transport activity, highlighting their involvement in cellular metabolism, protein synthesis, and oxygen delivery (Figure 5A).

KEGG pathway enrichment analysis revealed several pathways significantly associated with the transition of SHFs from the telogen-to-anagen phase. Notably, the ribosome pathway, directly associated with protein translation and synthesis, suggested heightened metabolic activity in anagen-phase SHFs. Additionally, the purine metabolism and riboflavin metabolism pathways reflected the increased demand for energy and nucleic acid metabolism to support SHF growth. The PPAR signaling and Notch signaling pathways, both well-known regulators of hair follicle cycling and cell differentiation, were also significantly enriched. Other enriched pathways, including complement and coagulation cascades, myocardial contraction, and tyrosine metabolism, may reflect the metabolic regulation and growth environment required for SHF development and regeneration (Figure 5B).

To further explore the key pathways and protein functions involved in the regulation of the SHF cycle in cashmere goats, we selected pathways related to hair follicle development from the KEGG enrichment analysis of the 458 DEPs and identified a subset of 28 key proteins (Supplementary Table S6). GO functional annotation and KEGG pathway enrichment analyses were subsequently performed on this protein set.

GO analysis showed that in the BP category, DEPs were significantly enriched in processes such as the positive regulation of signal transduction, Notch receptor processing, cell adhesion, and biological adhesion. These findings suggest that these proteins may promote the transition of telogen SHFs to anagen by regulating intercellular signaling and interactions. In the CC category, DEPs were mainly localized in intercellular junctions, protein kinase CK2 complexes, and *γ*-secretase complexes, implying that these proteins play an important role in intercellular signal exchange and molecular complex regulation. In the MF category, DEPs were significantly enriched in macromolecular complex binding and protein phosphatase activity, indicating their critical roles in protein modification and signal pathway regulation (Figure 5C).

KEGG analysis showed that DEPs were significantly enriched in several signaling pathways closely related to hair follicle development, growth, and regeneration. These included the Notch signaling pathway, Wnt signaling pathway, and Hippo signaling pathway, all of which are known to play central roles in hair follicle cycle regulation and stem cell activation. The PI3K-Akt signaling pathway and MAPK signaling pathway suggested that these proteins may be involved in initiating anagen by regulating cell proliferation and differentiation. ECM-receptor interaction and cytokine signaling pathways (such as TNF and Jak–STAT) further highlighted the importance of ECM remodeling and intercellular communication in the SHF microenvironment during the transition (Figure 5D).

In conclusion, an in-depth analysis of GO and KEGG indicates that the key DEPs involved in the telogen-to-anagen transition of SHFs are primarily associated with biological processes such as the positive regulation of signal transduction, cell adhesion, and protein modification. These proteins are significantly enriched in critical signaling pathways that regulate the hair follicle cycle. Collectively, these findings elucidate the functions and molecular mechanisms of key DEPs during the telogen-to-anagen transition in cashmere goat SHFs, providing valuable theoretical evidence for future research into SHF cycle regulation.

#### 3.2.3 Protein–protein interaction network analysis

**Figure 6:**
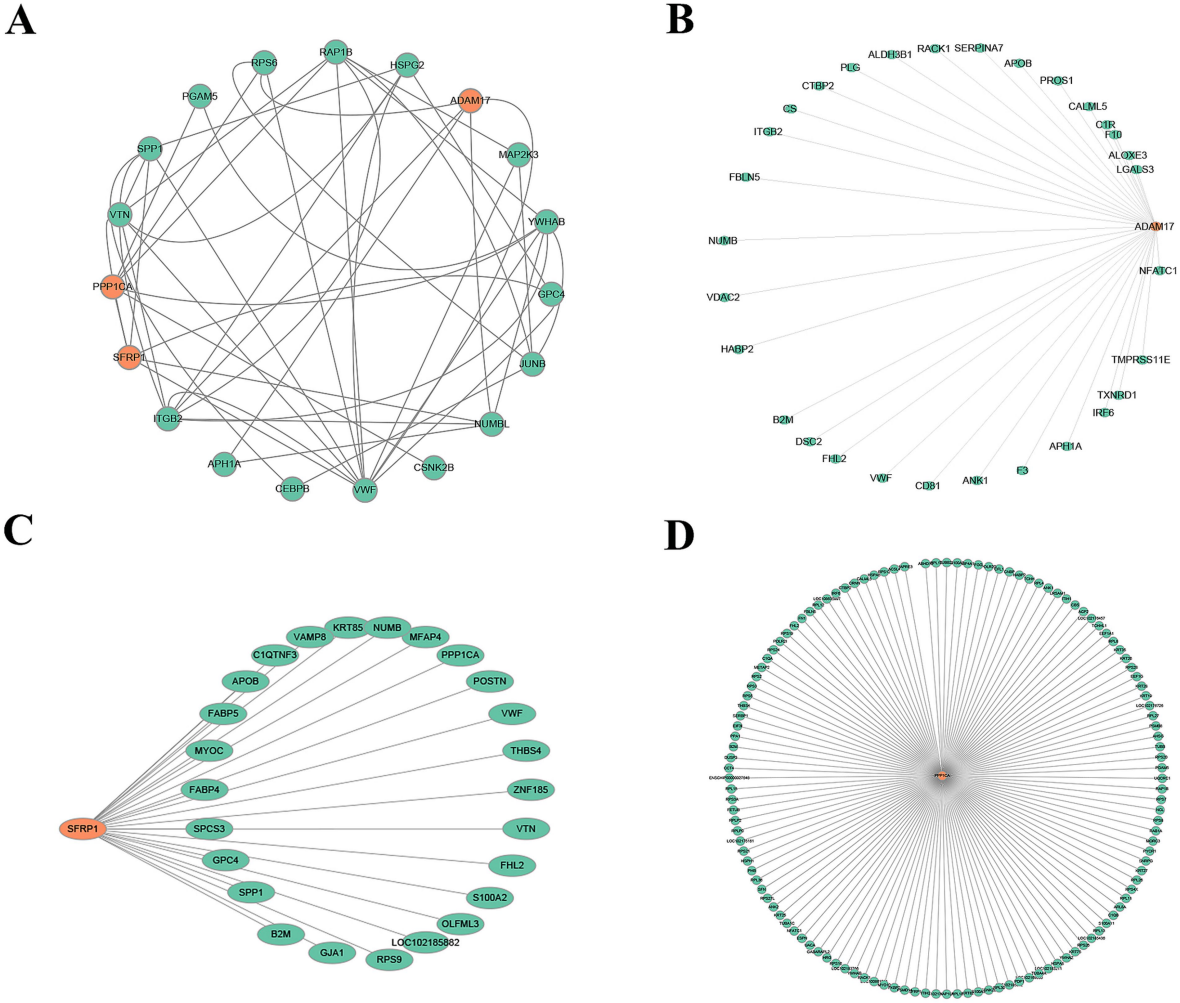
PPI networks. **(A)** Core PPI network of SFRP1, PPP1CA, and ADAM17. Orange nodes represent the target proteins (SFRP1, PPP1CA, and ADAM17), green nodes indicate proteins that directly interact with the target proteins, and gray edges represent the interactions between proteins. **(B)** Directed interaction network of ADAM17. The orange node represents ADAM17, green nodes indicate its directly interacting proteins, and gray edges represent the interaction relationships. The network highlights ADAM17’s role as a key regulatory factor interacting with multiple proteins. **(C)** Directed interaction network of SFRP1. The orange node represents SFRP1, green nodes indicate its interacting proteins, and gray edges represent the interaction relationships. The network suggests SFRP1’s potential involvement in ECM regulation and signal transduction. **(D)** Extensive interaction network of PPP1CA. The orange node represents PPP1CA, green nodes indicate its interacting proteins, and gray edges represent the interaction relationships. The network reveals PPP1CA’s potential roles in ribosomal protein regulation, signaling pathways, metabolism, and cytoskeleton organization.

To identify key factors involved in the periodic regulation of DEPs, a PPI network was constructed (Figure 6A). This network consisted of 19 nodes and multiple edges, representing the interactive relationships between proteins. Through topological analysis and functional annotation, three high-degree hub proteins—ADAM17, SFRP1, and PPP1CA—were identified as key regulators of the telogen-to-anagen transition in SHFs.

The interaction network of ADAM17 (Figure 6B) revealed its primary associations with ECM-related proteins, signal transduction-related proteins, metabolism- and oxidative stress-related proteins, and blood-related factors. This suggests that ADAM17 plays a critical role in remodeling the microenvironment of SHFs and mediating signaling pathways.

SFRP1 (Figure 6C) demonstrated close interactions with ECM-related proteins, metabolism-related proteins, signal transduction-related proteins, and cytoskeleton- and structure-related proteins. These findings indicate that SFRP1 is involved in regulating ECM signaling, maintaining structural integrity, and mediating metabolic processes.

The network of PPP1CA (Figure 6D) showed extensive interactions with ribosomal proteins, cytoskeleton-related proteins, signal regulation proteins, and metabolic proteins. This highlights PPP1CA’s role in modulating protein synthesis, cytoskeletal dynamics, and cellular signaling pathways.

Collectively, these findings suggest that ADAM17, SFRP1, and PPP1CA function as critical regulators in the telogen-to-anagen transition by influencing signal transduction, ECM remodeling, and metabolic processes. These results provide valuable insights into the molecular mechanisms underlying SHF cycle regulation.

### 3.3 Validation of ADAM17, SFRP1, and PPP1CA as key regulators during the telogen-to-anagen transition of SHFs

**Figure 7:**
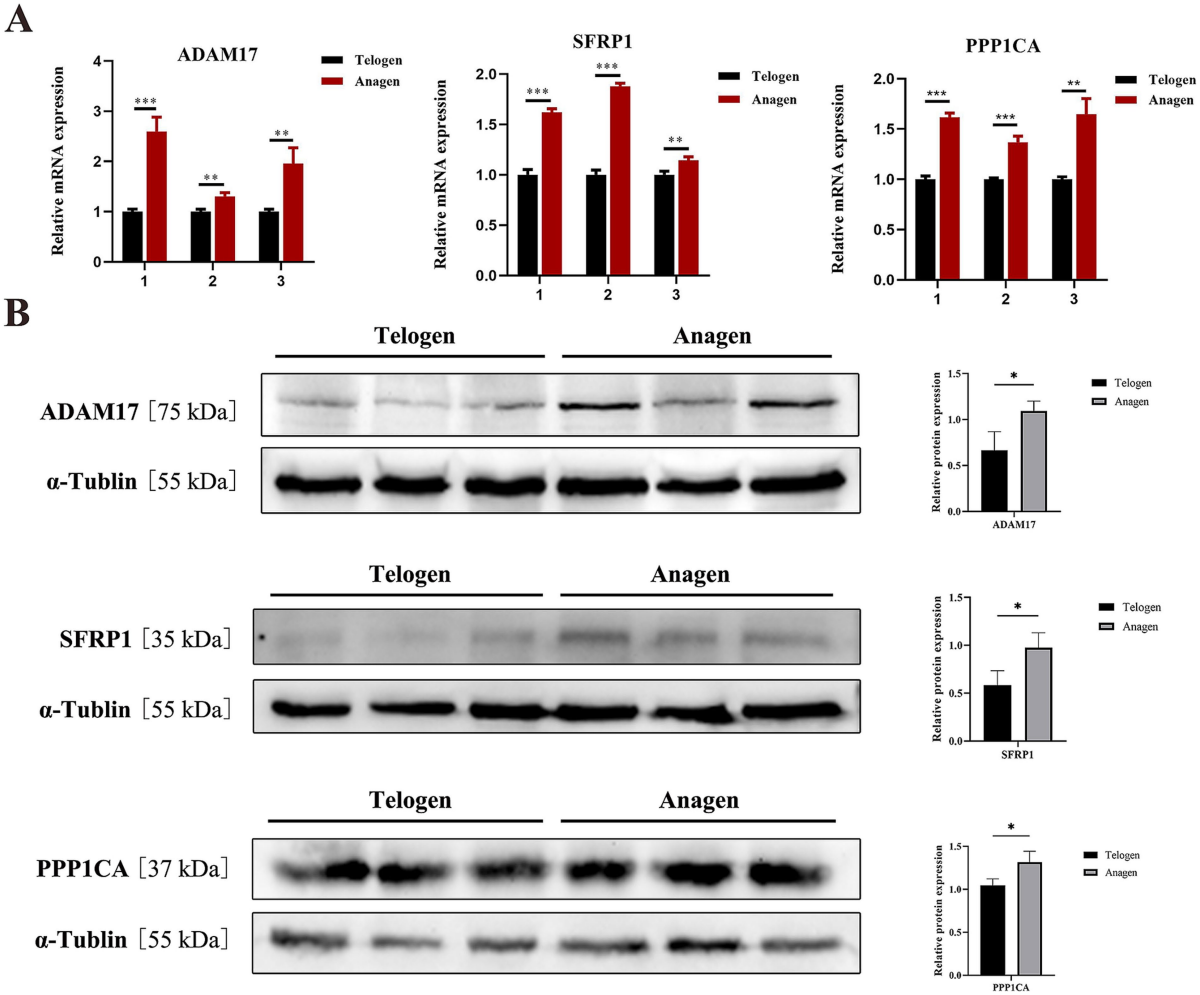
Validation of ADAM17, SFRP1, and PPP1CA as key regulators in the transition from telogen to anagen in SHFs. **(A)** Relative mRNA expression levels of ADAM17, SFRP1, and PPP1CA in telogen- and anagen-phase SHF samples, measured by RT-qPCR. The results show that the mRNA expression levels of all three genes are significantly higher in anagen samples compared to telogen samples. Samples 1, 2, and 3 represent skin tissues collected from the same three individual goats during the telogen and anagen phases (***p < 0.001; **p < 0.01). **(B)** Protein expression levels of ADAM17, SFRP1, and PPP1CA in telogen- and anagen-phase SHF samples, analyzed by Western blot with *α*-Tubulin as the internal control. Semi-quantitative analysis reveals that the protein expression levels of these three molecules are significantly higher in anagen samples than in telogen samples. Error bars represent the mean ± standard deviation (SD), with significance levels indicated as *p < 0.05.

RT-qPCR and Western blot analyses confirmed that the expression levels of ADAM17, SFRP1, and PPP1CA were upregulated during the telogen-to-anagen transition of SHFs (Figures 7A,B), consistent with the proteomic sequencing data. These findings suggest that these proteins may mediate the SHF cycle transition by regulating signal transduction and cell adhesion, providing a robust experimental foundation for further studies.

### 3.4 Immunolocalization of ADAM17, SFRP1, and PPP1CA in telogen and anagen phases of SHFs

**Figure 8:**
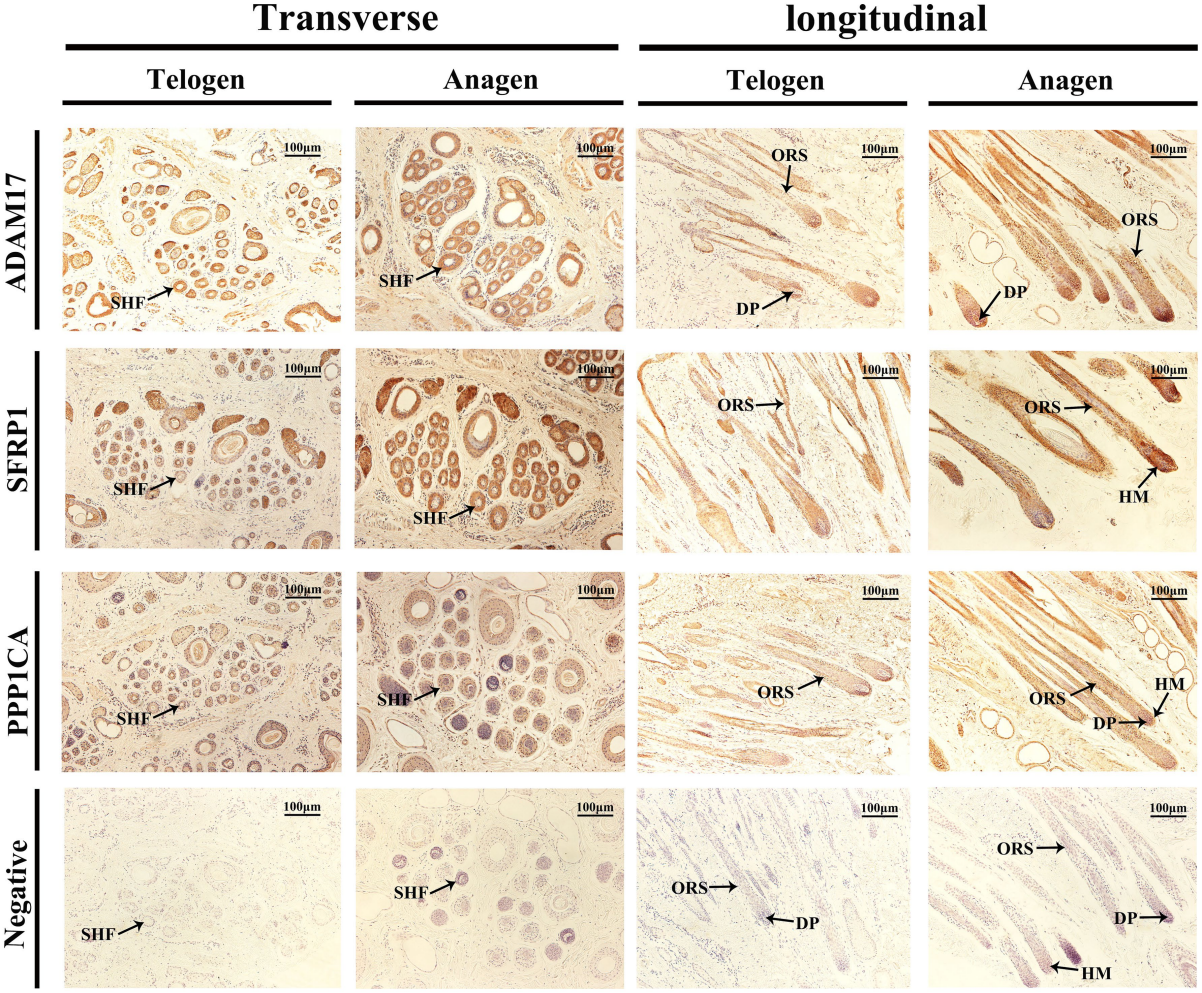
Immunolocalization of ADAM17, SFRP1, and PPP1CA in SHFs during the telogen and anagen phases. Immunohistochemical staining shows the expression and localization of ADAM17, SFRP1, and PPP1CA in transverse and longitudinal sections of SHFs from the dorsal region of cashmere goats during the telogen and anagen phases. Negative controls exhibit no staining, confirming the specificity of the antibodies. Scale bar: 100 μm.

Immunohistochemical staining was performed to analyze the expression and localization of ADAM17, SFRP1, and PPP1CA in SHFs from the dorsal region of cashmere goats during the telogen and anagen phases (Figure 8).

The immunolocalization and functional analysis of ADAM17 in transverse sections revealed its expression in SHFs during the telogen phase. During the anagen phase, its expression significantly increased in SHFs, particularly in the ORS. In longitudinal sections, ADAM17 exhibited weak expression in the ORS and DP of SHFs during telogen. However, its expression was markedly enhanced during anagen and localized primarily to the ORS and DP. These findings suggest that ADAM17 may facilitate the telogen-to-anagen transition by promoting ORS cells proliferation and mediating signal transduction in DPCs.

The immunolocalization and functional analysis of SFRP1 in transverse sections demonstrated its expression in SHFs during the telogen phase, which significantly increased during the anagen phase. Longitudinal sections revealed weak expression of SFRP1 in the ORS and HM of SHFs during the telogen phase. However, during the anagen phase, its expression was markedly enhanced and primarily localized to the ORS and HM of SHFs. As an antagonist of the Wnt signaling pathway, SFRP1 likely supports SHF growth and regeneration by modulating the activity of SHFSCs and promoting the differentiation of HMCs.

The immunolocalization and functional analysis of PPP1CA in transverse sections revealed its expression in SHFs, with significantly heightened levels during the anagen phase, predominantly localized to the ORS. Longitudinal sections demonstrated weak expression of PPP1CA in the ORS during the telogen phase. However, during the anagen phase, its expression markedly increased and expanded to the ORS, HM, and DP. As a phosphatase, PPP1CA may promote the proliferation and differentiation of ORS cells, HMCs, and DPCs in SHFs by regulating the phosphorylation of cell cycle regulators and signaling components.

In summary, immunohistochemical analysis demonstrated dynamic and differential expression of ADAM17, SFRP1, and PPP1CA in hair follicles during the telogen and anagen phases, with primary localization to key regions such as the ORS, DP, and HM. These proteins likely regulate the SHF cycle by modulating signal transduction, stem cell activity, and cell proliferation. These findings provide important evidence for further exploration of the molecular mechanisms underlying SHF regulation.

## 4 Discussion

Hair follicles are important miniature organs in the skin, composed of both epidermal and dermal components. Their primary function is to produce hair through periodic activity, playing a vital role in thermoregulation, skin barrier formation, and environmental sensing in animals (21). The hair follicle cycle is divided into the anagen, catagen, and telogen. This dynamic periodicity is essential for hair growth, shedding, and regeneration (22, 23). The normal maintenance of the hair follicle cycle relies on a complex molecular signaling network, which is not only significant for understanding the regulatory mechanisms of the SHF cycle but also provides a theoretical basis for improving cashmere quality in cashmere goats.

Proteins, as pivotal molecules in cell signaling and functional regulation, play a central role in orchestrating transitions between different phases of the SHF cycle. Previous studies have primarily focused on the regulation of the hair follicle cycle by pathways such as Wnt, Notch, and Jak–STAT (2, 8, 16, 24). However, systematic studies on the functions, dynamic expression patterns, and mechanisms of specific proteins during different phases of the SHF cycle remain limited. While this study is constrained by a small sample size, we minimized individual variability by collecting telogen and anagen skin samples from the same cashmere goat. Using proteomics, we successfully identified key proteins associated with the SHF cycle. This high-throughput proteomic approach provides a rapid, efficient, and comprehensive method to systematically analyze the dynamic changes in proteins during the SHF cycle, offering new insights into the molecular mechanisms underlying SHF regulation (25).

In this study, we performed LC–MS/MS-based proteomic analysis on skin tissues from telogen and anagen phases in cashmere goats, revealing dynamic protein changes during the telogen-to-anagen transition of SHFs. A large number of proteins were found to be either upregulated or downregulated, indicating significant protein remodeling during this phase, which is likely associated with the reconstruction of the non-permanent region of SHFs. Reconstruction of the non-permanent region is a critical turning point in hair follicle development and marks the initiation of the telogen-to-anagen transition (26). This event involves a series of complex biological processes, including cell proliferation and differentiation, ECM remodeling, signaling pathway regulation, and the activation of HFSCs (27–30). During this process, HFSCs must first transition from a quiescent to an active state, followed by amplification and differentiation to form new hair follicle structures (28). Concurrently, ECM remodeling provides mechanical support and signaling cues for hair follicle growth. Growth factors and cytokines play key roles in regulating HFSCs activation through signaling pathways (31–33). These mechanisms ensure the structural integrity and functional restoration of hair follicles. Additionally, HFSCs activation is tightly regulated by DP signals. As the “regulatory center” of the hair follicle cycle, the DP communicates bidirectionally with HFSCs by secreting signaling molecules, thereby promoting hair follicle regeneration (15).

Our findings revealed that ADAM17 was significantly upregulated during the anagen phase, suggesting that it may facilitate the activation of the telogen-to-anagen transition by cleaving membrane-bound growth factors. ADAM17 is a “sheddase” that cleaves membrane-bound proteins to release their active forms, such as epidermal growth factor (EGF) and tumor necrosis factor-*α* (TNF-α) (34–37). Evidence suggests that EGFR ligands, such as EGF, promote the proliferation and migration of ORS cells (38–41). Additionally, the EGFR ligand EREG activates ORS cells and HMCs through EGFR signaling (42). These growth factors are critical for SHFSCs activation and hair follicle regeneration. In cashmere goats, EGFR signaling may play a central role in regulating SHFSCs proliferation and migration, laying the foundation for cashmere fiber formation. EGFR is primarily expressed in the ORS and DP of SHFs, and immunohistochemical analysis showing ADAM17 localization in these regions further supports its role in epithelial-mesenchymal interactions, which are central to SHFs cycle regulation. Previous studies have shown that EGFR-mediated signaling not only promotes HFSC amplification but also maintains the dynamic balance of the hair follicle microenvironment by regulating inflammation and ECM remodeling (43, 44).

As an antagonist of the Wnt signaling pathway, SFRP1 was highly expressed in the ORS and HM during the anagen phase, indicating that it may finely regulate Wnt signaling to balance SHFSCs activation and differentiation. The Wnt signaling pathway plays a dual role in hair follicle regulation: it can activate *β*-catenin to promote stem cell proliferation or, under certain conditions, inhibit differentiation to maintain stem cell quiescence (45, 46). The high expression of SFRP1 may act as a “regulator,” partially suppressing Wnt signaling to prevent excessive stem cell differentiation, thereby maintaining the normal hair follicle growth cycle (47). This mechanism is particularly important in cashmere goats, as the periodic renewal of SHFs directly determines the growth quality and length of cashmere fibers. Interestingly, the role of SFRP1 in cashmere goat hair follicles differs from studies in mouse models, where SFRP1 was reported to inhibit Wnt-driven hair follicle activation (48). This discrepancy may reflect species-specific differences in hair follicle biology or the unique developmental requirements of SHFs in cashmere goats. Further research into the regulation of Wnt signaling by SFRP1 across species will help elucidate its universal and specific roles in hair follicle cycle regulation.

PPP1CA, a phosphatase involved in cell cycle regulation, was prominently expressed in the ORS, DP, and HM during the anagen phase. This expression pattern suggests a role in maintaining SHFSCs proliferation and differentiation, consistent with its role in promoting glioblastoma growth (49). PPP1CA facilitates the G1/S transition of the cell cycle by dephosphorylating retinoblastoma protein, thereby accelerating cell proliferation (50). Additionally, PPP1CA may regulate the activity of cell cycle-related proteins such as c-Myc (51, 52), supporting the rapid proliferation of HMCs to meet the demands of rapid SHFs growth during the anagen phase. Given the DP’s role as the “signaling center” of hair follicle growth, the high expression of PPP1CA in the DPCs may promote SHFSCs activation and differentiation by regulating the secretion of signaling molecules.

In conclusion, ADAM17, SFRP1, and PPP1CA likely work in concert to regulate the telogen-to-anagen transition and promote cashmere fiber production. ADAM17 activates SHFSCs through the EGFR signaling pathway; SFRP1 balances Wnt signaling to regulate stem cell activation and promoting the differentiation of HMCs; and PPP1CA promotes SHF cell proliferation by modulating phosphorylation states. The precise spatial and temporal regulation of these proteins collectively drives the periodic renewal of cashmere goat SHFs.

Despite the significant findings of this study, several limitations remain. First, the small sample size may limit the generalizability of the results. Second, although the expression patterns of ADAM17, SFRP1, and PPP1CA were characterized, their functions were not validated through gene knockout or overexpression experiments. Future studies should focus on *in vivo* and *in vitro* functional analyses to elucidate the specific mechanisms of these molecules. Additionally, exploring the upstream regulators and downstream targets of these proteins will further enhance our understanding of their roles in hair follicle biology.

## 5 Conclusion

This study, through the integration of morphological analysis, proteomics, and experimental validation, uncovered dynamic changes during the telogen-to-anagen transition in cashmere goat SHFs. Furthermore, it established a connection between key DEPs—ADAM17, SFRP1, and PPP1CA—and the regulation of the SHF cycle. These findings not only deepen our understanding of the molecular mechanisms regulating the SHF cycle but also highlight potential molecular targets for enhancing cashmere fiber quality.

## Data Availability

The mass spectrometry proteomics data have been deposited to the ProteomeXchange Consortium via the PRIDE partner repository with the dataset identifier PXD060635.
