# Supplementary material for: Comparative proteomic analysis of the telogen-to-anagen transition in cashmere goat secondary hair follicles
Source: Front Vet Sci. 2025 Feb 25;12:1542682. doi: 10.3389/fvets.2025.1542682 (PMC11894581; doi:10.3389/fvets.2025.1542682)
Supplement: Supplementary file 2 [file Table_2.pdf]

Supplementary Table S2. RT-qPCR Primer Information

| Gene Name | Sequence (5'-3') | Length (bp) |
|---|---|---|
| GAPDH | F: TTCCACGGCACAGTCAAGG<br>R: CTCAGCACCAGCATCACCC | 114 |
| ADAM17 | F—CGCAGCAACAAAGTATGTGGGA<br>R—CATCGGCGGCATTTCCAG | 92 |
| SFRP1 | F—GCTCAAGTGCGACAAGTTTCC<br>R—TCGATTATGGCTTCCGATTT | 87 |
| PPP1CA | F—TGGTCTGTGCCTCAAATCCC<br>R—CCGTGAATGTCACCGCAGAT | 93 |
