# Supplementary material for: Comparative proteomic analysis of the telogen-to-anagen transition in cashmere goat secondary hair follicles
Source: Front Vet Sci. 2025 Feb 25;12:1542682. doi: 10.3389/fvets.2025.1542682 (PMC11894581; doi:10.3389/fvets.2025.1542682)
Supplement: Supplementary file 3 [file Table_3.pdf]

Supplementary Table S3. Information of Antibodies Used in This Study for WB Assays.

Antibodies	Source	Identifier	Host	Proportion
Anti-ADAM17	Proteintech	29948-1-AP	Rabbit	1:5000
Anti-SFRP1	Proteintech	26460-1-AP	Rabbit	1:5000
Anti-PPP1CA	Proteintech	28617-1-AP	Rabbit	1:5000
